# A damage constitutive model of layered slate under the action of triaxial compression and water environment erosion

**DOI:** 10.1038/s41598-024-58872-4

**Published:** 2024-04-10

**Authors:** Jian Jia, Tiejun Tao, Xingchao Tian, Caijin Xie, Bingxi Jian, Guoqing Li

**Affiliations:** 1 College of Mining, Guizhou University, Guiyang, 550025 China; 2 Guizhou Power Transmission and Transformation Co., Ltd, Guiyang, 550002 China; 3 College of Civil Engineering, Guizhou University, Guiyang, 550025 China

**Keywords:** Bedding slate, Triaxial compression, Transversely isotropic, Deformation elements, Damage constitutive model, Civil engineering, Geology

## Abstract

Based on the macroscopic structure control theory, The slate with a significant bedding plane is a composite rock mass composed of rock blocks containing microscopic defects, joint surface closure elements, and shear deformation elements. Considering the coupling damage effect of water erosion and triaxial compressive load on bedding structure plane, the transversely isotropic damage constitutive model of slate under triaxial compressive load is derived with the dip angle of bedding and confining pressure as the variable. Firstly, based on the statistical theory of continuous damage mechanics and the maximum tensile strain criterion, the transversely isotropic deformation constitutive model of rock block with micro-defects is given; Secondly, based on the phenomenological theory of closed deformation and shear-slip deformation mechanism of layered structural plane under the coupling action of water erosion and triaxial compression load, the calculation formula of axial deformation of layered structural plane under the coupling action is given; Finally, to verify the accuracy of the established constitutive model, triaxial compression tests are carried out to study the influence of dip angle and confining pressure on the macroscopic mechanical properties and mechanism of slate. The results show that: the established triaxial compression damage constitutive model of bedding slate can accurately describe the stress–strain relationship of bedding slate after water environment erosion. With the increase of bedding dip angle, the strength and deformation capacity of the bedding slate first decreases and then increases, showing a U-shaped distribution as a whole. There are three main types of failure: tension shear composite failure, shear slip failure, and splitting tension failure.

## Introduction

Various anisotropic rocks, such as slate, limestone, and shale, are often encountered in the excavation of underground projects such as tunnels^1–3^. Among them, the dip angle of the stratigraphic plane of slab rock is the key factor affecting the macroscopic properties of rock mass mechanics and the stability of surrounding rock. In the water-rich environment, the mineral composition of the rock mass changes after the rock mass is eroded by groundwater, and the microstructure is damaged to varying degrees, resulting in the macroscopic mechanical properties of the rock mass being affected^4–6^. In the neighborhood of rock engineering, theoretical analysis, laboratory experiments, numerical simulation, and other methods are usually used to explore the mechanical properties and damage mechanisms of rock. The mechanical properties of slate are a hot topic^7–15^. It is of great theoretical value and engineering guiding significance to consider the influence of the bedding dip angle of slate and the erosion of the water environment on the mechanical properties and damage development law of slate.

The test results of many indoor rock samples show that slate belongs to metamorphic rock, with obvious bedding structure, developed fissures, softening in water, and significant transverse isotropy. Based on uniaxial compression test, triaxial compression test, brazilian test, and set-up for wave velocity measurement, Alejano et al.^16,17^ studies the failure mechanism and mechanical properties of transversely isotropic rocks, gives an experimental determination method of five elastic parameters in the transversely isotropic elastic model, and verifies its reliability and applicability. Dambly et al.^18^ proposed a method to directly measurement the shear moduli of transversely isotropic rock mass by uniaxial compression test. Through uniaxial, triaxial, and brazilian tests, Gholami^19^ studied the influence of the change of bedding dip angle on the strength and deformation capacity of slate under wet and dry conditions. Ding et al.^20^ studied the influence of bedding inclination on the mechanical properties of slate through a uniaxial compression test, observed the morphology of fracture surface through scanning electron microscope (SEM), and explained its fracture mode and micro failure mechanism. Xu et al.^21^ carried out uniaxial compressive creep test combined with particle discrete element method simulation to study the creep behavior of phyllite under different water content. Chen et al.^22^ carried out triaxial compression test to study the influence of bedding dip angle and water content on the anisotropic mechanical behavior of phyllite, using the temporal and spatial distributions of the AE counts acoustic emission experiments to study the process of crack propagation and propagation to failure, the morphology and roughness of the fracture surface were analyzed by scanning electron microscope (SEM).

In theoretical research, based on continuum damage mechanics, fracture mechanics theory, and damage statistical distribution law, many scholars have established a variety of damage constitutive models^23–36^, and their research content mainly includes four aspects: (1) Select the failure strength criteria of rock microelements, such as maximum principal strain, Drucker–Prager, Hoek–Brown, and Mohr–Coulomb criterion; (2) Determine the distribution law of rock microelement strength, such as power function distribution, Weibull distribution, and lognormal distribution; (3) The determination methods of statistical parameters, such as linear fitting method, inverse analysis method, and peak point method; (4) The accuracy and applicability of the model are verified by indoor experiments and numerical simulation. Based on the Drucker–Prager criterion, Chen^28^ proposed a statistical damage constitutive model of triaxial compression of acid etched rock under the coupling effect of temperature and confining pressure. Based on the Hoek–Brown criterion, Chen^29^ established a constitutive model of rock triaxial compression damage, which can better predict the strain softening characteristics of rock and has a simple expression. Based on the modified Hoek–Brown criterion, Zhou^30^ established a statistical damage constitutive model of rock considering the influence of joint inclination. Fu^31^ proposed a statistical damage constitutive model that can accurately predict the triaxial compressive strength of anisotropic rocks, which is related to the number of thawing cycles, confining pressure, and bedding direction. Li et al.^24,32^ considered the strain softening phenomenon of materials and the influence of intermediate principal stress and established the triaxial compression damage constitutive model of soft rock. Liu^33^ established the triaxial compression damage constitutive model of layered composite rock and determined the model parameters by the curve fitting method.Based on the fracture theory and Weibull distribution, Liu^34^ established a damage constitutive model of jointed rock mass with micro defects and macro coupling under uniaxial compression. Liu^35^ proposed a damage constitutive model of non-penetrating jointed rock under biaxial compressive load, considering the influence of joint inclination and confining pressure. Based on the Drucker–Prager criterion, Chen^36^ established a coupled damage constitutive model of non-penetrating jointed rock mass, which considered the crack propagation length and joint friction effect. Wang^37^ considered the closure and shear deformation of the bedding structural plane and proposed a mathematical model that can simulate the complete stress–strain curve of a multi-jointed rock mass. Liu^38^ deduced the micro defects and macro coupling damage variables and established the damage statistical constitutive model of rock through the joint plane, which considered the shear strength of joints and the influence of microcracks on the strength and deformation capacity of a rock mass. Yang et al.^39–41^ Believed that the rock deformation with significant bedding structure should include the deformation of the joint surface. Taking into account the micro defects of rock blocks and the deformation of the joint surface, they proposed a static constitutive model, which simulates the rock with a significant bedding structure by connecting the rock block damage body with micro defects in series with the bedding deformation element.

The research on the damage constitutive model of slate mainly focuses on the influence of failure strength criterion and bedding dip angle on mechanical properties. Many results show that the transversely isotropic damage constitutive model is suitable for bedding slate. For slate in a water-rich environment, few damage constitutive models characterize the influence of bedding dip angle and confining pressure on the macroscopic mechanical properties of bedding slate under triaxial compression load. Based on the structural control theory, the influence of bedding dip angle and confining pressure on the macroscopic mechanical properties and damage evolution of anisotropic slate is considered in this paper. In Cartesian coordinates, the elastic constitutive equation of transversely isotropic is adopted to equivalent bedding slate, and its independent elastic parameters are changed from 2 to 5 when isotropic. Based on phenomenological theory, it is considered that the damage to bedding structure plane under compressive load includes the initial damage caused by water erosion before loading and the damage caused by deformation and instability of bedding during loading. Based on the calculation equations of closure deformation and shear slip deformation of layered structural plane under triaxial compression load, an effective and simple mathematical expression is established to characterize the stress–strain curve of layered slate under triaxial compression load after water erosion. In addition, to verify the accuracy and reliability of the established damage constitutive model and to analyze the influence of changes in confining pressure and bedding on the damage evolution process, mechanical properties, and failure mechanism of slate specimens, triaxial compression tests of laminated slate specimens with different dip angles and confining pressures carried out.

## Damage evolution equation

**Figure 1:**
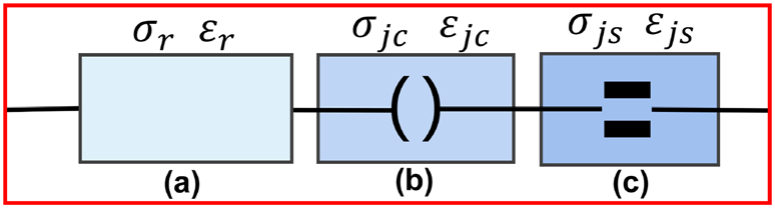
Layered slate deformation element combination model diagram. (**a**) Rock blocks containing micro defects; (**b**) Closed deformation elements; (**c**) Shear slip deformation elements.

Existing research shows that the significant slate deformation of bedding structures should include rock block deformation, closure deformation of the bedding structural plane, and shear slip deformation^39,41^. Based on the theory of rock mass structure, this paper uses rock blocks containing micro defects, closed deformation elements of bedding discontinuities, and shear slip deformation elements in series to simulate the deformation model of laminated slate^11,41^. To establish a relatively complete structural mechanics system, as shown in Fig. 1.

The axial stress and strain of the bedding slate specimen under triaxial compression load are $\sigma_{z}$ and $\varepsilon_{z}$, respectively. The combination relationship shown in Fig. 1, It can be expressed as Eq. (1)

1 $$ \left\{ {\begin{array}{*{20}l} {\sigma_{z} = \sigma_{r} = \sigma_{jc} = \sigma_{js} } \hfill \\ {\varepsilon_{z} = \varepsilon_{r} + \varepsilon_{jc} + \varepsilon_{js} } \hfill \\ \end{array} } \right. $$

In Eq. (1), $\sigma_{r}$ is the axial stress of rock block, $\sigma_{jc}$ is the axial stress of joint closure deformation, and $\sigma_{js}$ is the axial stress of joint shear deformation; $\varepsilon_{r}$ is the axial strain of rock block, $\varepsilon_{jc}$ is the axial strain during joint closure deformation, and $\varepsilon_{js}$ is the axial strain during joint closure deformation.

The main task of establishing a damage constitutive model is to give mathematical expressions that accurately describe the macroscopic mechanical behavior and damage evolution of three deformation elements under triaxial compression. The subsequent contents of this section give the damage evolution equations of the two deformation elements of the rock damage body and the bedding structural plane under load and solve them according to the combination of Eq. (1).

### Damage variable of rock blocks containing micro defects

#### Constitutive model of transversely isotropic elastic

**Figure 2:**
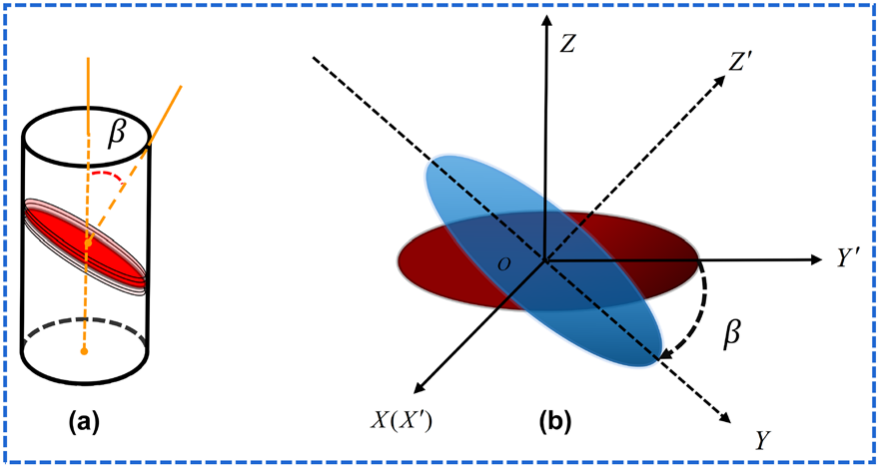
Diagram of relationship between joint dip angle and coordinate. (**a**) Definition of anisotropy angle *β*; (**b**) Local $X^{\prime}Y^{\prime}Z^{\prime}$ and global XYZ coordinate systems.

When the bedding structure plane of the slate is stable, it is feasible to analyze the rock block with a transversely isotropic elastic constitutive model^16^. Establish the coordinate system as shown in Fig. 2. The global coordinate system ${\text{XYZ}}$ rotates clockwise around the X axis to form a local coordinate system ${\text{X}}^{\prime } {\text{Y}}^{\prime } {\text{Z}}^{\prime }$. The angle between the normal direction of the isotropic surface (joint dip structure surface) and the Z axis is the anisotropy angle *β*(the bedding dip angle).

In the Cartesian coordinate system, the generalized Hooke’s law can be used to describe the transversely isotropic elastic constitutive relation of slate, and its independent elastic parameters are reduced from 21 to 5 in the case of extreme anisotropy. In the local coordinate system, the stress–strain relationship is as follows^17,42^:

2 $$ \varepsilon^{\prime}_{r} = S^{\prime}\sigma^{\prime}_{r} $$

In Eq. (2), $\varepsilon^{\prime}_{r}$ is the strain tensor; $\sigma^{\prime}_{r}$ is the stress tensor; $$S^{\prime} = \left[ {\begin{array}{*{20}c} A & 0 \\ 0 & B \\ \end{array} } \right]$$ is the elastic flexibility matrix, where $$A = \left[ {\begin{array}{*{20}c} {{{1} \mathord{\left/ {\vphantom {{1} {E_{1} }}} \right. \kern-0pt} {E_{1} }}} & {{{ - v_{1} } \mathord{\left/ {\vphantom {{ - v_{1} } {E_{1} }}} \right. \kern-0pt} {E_{1} }}} & {{{ - v_{2} } \mathord{\left/ {\vphantom {{ - v_{2} } {E_{{2}} }}} \right. \kern-0pt} {E_{{2}} }}} \\ {{{ - v_{1} } \mathord{\left/ {\vphantom {{ - v_{1} } {E_{1} }}} \right. \kern-0pt} {E_{1} }}} & {{{1} \mathord{\left/ {\vphantom {{1} {E_{1} }}} \right. \kern-0pt} {E_{1} }}} & {{{ - v_{2} } \mathord{\left/ {\vphantom {{ - v_{2} } {E_{{2}} }}} \right. \kern-0pt} {E_{{2}} }}} \\ {{{ - v_{2} } \mathord{\left/ {\vphantom {{ - v_{2} } {E_{{2}} }}} \right. \kern-0pt} {E_{{2}} }}} & {{{ - v_{2} } \mathord{\left/ {\vphantom {{ - v_{2} } {E_{{2}} }}} \right. \kern-0pt} {E_{{2}} }}} & {{{1} \mathord{\left/ {\vphantom {{1} {E_{{2}} }}} \right. \kern-0pt} {E_{{2}} }}} \\ \end{array} } \right]$$ and $B = diag\left[ {{{2(1 + v_{1} )} \mathord{\left/ {\vphantom {{2(1 + v_{1} )} {E_{1} }}} \right. \kern-0pt} {E_{1} }},{{1} \mathord{\left/ {\vphantom {{1} {G_{2} ,{{1} \mathord{\left/ {\vphantom {{1} {G_{2} }}} \right. \kern-0pt} {G_{2} }}}}} \right. \kern-0pt} {G_{2} ,{{1} \mathord{\left/ {\vphantom {{1} {G_{2} }}} \right. \kern-0pt} {G_{2} }}}}} \right]$. In the matrix, $E_{1}$ and $v_{1}$ are the elastic modulus and Poisson’s ratio in the isotropic plane, respectively; $E_{{2}}$, $v_{2}$ and $G_{2}$ represent the elastic modulus, Poisson’s ratio, and shear modulus perpendicular to the isotropic plane, respectively.

Similarly, the stress–strain relationship of a transversely isotropic body in the global coordinate system XYZ is as follows:

3 $$ \varepsilon_{r} = S\sigma_{r} $$

4 $$ S = \left[ {\begin{array}{*{20}c} {S_{11} } & {S_{{1{2}}} } & {S_{{1{3}}} } & {S_{{1{4}}} } & {S_{{1{5}}} } & {S_{{1{6}}} } \\ {S_{{{2}1}} } & {S_{{{22}}} } & {S_{{{23}}} } & {S_{{{24}}} } & {S_{{{25}}} } & {S_{{{26}}} } \\ {S_{{{3}1}} } & {S_{{{32}}} } & {S_{{{33}}} } & {S_{{{34}}} } & {S_{{{35}}} } & {S_{{{36}}} } \\ {S_{{{41}}} } & {S_{{{42}}} } & {S_{{{43}}} } & {S_{{{44}}} } & {S_{{{45}}} } & {S_{{{46}}} } \\ {S_{{{5}1}} } & {S_{{{52}}} } & {S_{{{53}}} } & {S_{{{53}}} } & {S_{{{55}}} } & {S_{{{56}}} } \\ {S_{{{61}}} } & {S_{{{62}}} } & {S_{{{63}}} } & {S_{{{64}}} } & {S_{{{65}}} } & {S_{{{66}}} } \\ \end{array} } \right] $$

In Eqs. (3): $\varepsilon_{r}$ represents the strain tensor, $\sigma_{r}$ refers to stress tensor; *S* is the elastic flexibility matrix, and each component can be obtained by the transformation Eq. (5) between the local coordinate system and the global coordinate system.

5 $$ S = R^{T} S^{\prime}R $$

In Eq. (5), R is the coordinate transformation matrix, which can be expressed as:

6 $$ R = \left[ {\begin{array}{*{20}c} 1 & 0 & 0 & 0 & 0 & 0 \\ 0 & {\cos^{2} \beta } & {\sin^{2} \beta } & 0 & { - \sin (2\beta )} & 0 \\ 0 & {\sin^{2} \beta } & {\cos^{2} \beta } & 0 & {\sin (2\beta )} & 0 \\ 0 & 0 & 0 & {\cos \beta } & 0 & {\sin \beta } \\ 0 & {\sin (2\beta )/2} & { - \sin (2\beta )/2} & 0 & {\cos (2\beta )} & 0 \\ 0 & 0 & 0 & {\sin \beta } & 0 & { - \cos \beta } \\ \end{array} } \right] $$

Through Eqs. (5) and (6), the elastic flexibility matrix components required in this paper are obtained, such as Eqs. (7)–(9):

7 $$ S_{{{13}}} = S_{{{31}}} = - \frac{{v_{1} }}{{E_{1} }}{\text{sin}}^{{2}} \beta - \frac{{v_{2} }}{{E_{2} }}\cos^{{2}} \beta $$

8 $$ S_{{{23}}} = S_{{{32}}} = \frac{{{\text{sin}}^{{2}} (2\beta )}}{4}\left( {\frac{1}{{E_{1} }} + \frac{1}{{E_{2} }} - \frac{1}{{G_{2} }}} \right) - \frac{{v_{2} }}{{E_{2} }}{\text{(sin}}^{{4}} \beta + \cos^{{4}} \beta ) $$

9 $$ S_{{{33}}} = \frac{{{\text{sin}}^{{2}} (2\beta )}}{4}\left( {\frac{1}{{G_{2} }} - \frac{{2v_{2} }}{{E_{2} }}} \right) + \frac{{\cos^{{4}} \beta }}{{E_{2} }} + \frac{{{\text{sin}}^{{4}} \beta }}{{E_{1} }} $$

The elastic parameters in the elastic flexibility matrix of rock mass are inherent properties^18^. The five elastic parameters in the above elastic flexibility matrix are inherent properties of slate, and the theoretical values of the five elastic parameters of transversely isotropic rock block can be determined by the triaxial compression test of slate specimens. Slate samples with joint dip angles of 0°, 45° and 90° are selected for static compression test to determine five independent elastic parameters. In Table 1, the calculation Eqs. (10)–(18) are derived from Eqs. (4)–(9).

**Table 1:** Five elastic determination equations of transversely isotropic slate.

The joint dip angle *β*(°)	Calculating equations
$\beta = {0}^\circ$	$\frac{{\Delta \varepsilon_{x} }}{{\Delta \sigma_{r} }} = - \frac{{v_{2} }}{{E_{{2}} }}$ (10)
	$$\frac{{\Delta \varepsilon_{y} }}{{\Delta \sigma_{z} }} = - \frac{{v_{2} }}{{E_{{2}} }}$$ (11)
	$$\frac{{\Delta \varepsilon_{r} }}{{\Delta \sigma_{r} }} = \frac{1}{{E_{{2}} }}$$ (12)
$\beta = {90}^\circ$	$\frac{{\Delta \varepsilon_{x} }}{{\Delta \sigma_{r} }} = - \frac{{v_{{1}} }}{{E_{{1}} }}$ (13)
	$$\frac{{\Delta \varepsilon_{y} }}{{\Delta \sigma_{r} }} = - \frac{{v_{2} }}{{E_{{2}} }}$$ (14)
	$$\frac{{\Delta \varepsilon_{r} }}{{\Delta \sigma_{r} }} = \frac{1}{{E_{{1}} }}$$ (15)
$\beta = {45}^\circ$	$\frac{{\Delta \varepsilon_{x} }}{{\Delta \sigma_{r} }} = - \frac{{1}}{{2}}\left( {\frac{{v_{{1}} }}{{E_{{1}} }} + \frac{{v_{{2}} }}{{E_{{2}} }}} \right)$ (16)
	$$\frac{{\Delta \varepsilon_{y} }}{{\Delta \sigma_{r} }} = \frac{{1}}{4}\left( {\frac{1}{{E_{1} }} + \frac{1}{{E_{2} }} - \frac{1}{{G_{2} }}} \right) - \frac{{v_{2} }}{{{2}E_{2} }}$$ (17)
	$$\frac{{\Delta \varepsilon_{r} }}{{\Delta \sigma_{r} }} = \frac{{1}}{4}\left( {\frac{{1}}{{E_{2} }} + \frac{{1}}{{E_{1} }} + \frac{1}{{G_{2} }} - \frac{{2v_{2} }}{{E_{2} }}} \right)$$ (18)

#### Damage evolution equation of rock blocks containing micro defects

Based on the theory of statistical damage mechanics, it is considered that the micro defects in the layered slate sample can be regarded as random damage, and its internal damage development has randomness and complexity under triaxial compression load^29^. The joint slate is composed of numerous micro-elements, and the macroscopic mechanical properties are the comprehensive reaction of many micro-element combinations. Assuming that the undamaged part of the micro-element bears the load, ^38,43^ defining the damage variable $D_{m}$ as the ratio of the number of micro-elements of damage failure $N_{f}$ to the total number of micro-elements $N_{t}$, and the statistical damage variable is expressed as Eq. (19).

19 $$ D_{m} = \frac{{N_{f} }}{{N_{t} }} $$

The strength of micro elements obeys the maximum tensile strain failure criterion, and the damage obeys the random distribution and has statistical rules. The loading process of rock block material is a continuous damage process, and the Weibull distribution function^30^ can be used to quantify the damage degree, as Eq. (20):

20 $$ P\left( {\varepsilon_{r} } \right) = \left\{ {\begin{array}{*{20}l} {\frac{m}{{F_{0} }}\left( {\frac{{\varepsilon_{{\text{r}}} }}{{F_{0} }}} \right)^{m - 1} \exp \left[ { - \left( {\frac{{\varepsilon_{{\text{r}}} }}{{F_{0} }}} \right)^{m} } \right]{\kern 1pt} ,} \hfill & {\varepsilon_{r} > {0}} \hfill \\ {0,} \hfill & {\varepsilon_{r} \le {0}} \hfill \\ \end{array} } \right. $$

In Eq. (20), $P\left( {\varepsilon_{r} } \right)$ is the probability density function, and $\varepsilon_{r}$ is the random distribution variable of micro-element strength; m and *F*_*0*_ are the parameters of the Weibull distribution that reflect the degree of concentration and the strength magnitude of the micro-elements, respectively^34^. With the increase of load, when the strain of the rock sample reaches a certain degree, the statistical expression of the number of rock damage failure micro-elements is Eq. (21).

21 $$ N_{f} (\varepsilon_{r} ) = \int_{0}^{\varepsilon } {N_{t} } P\left( {\varepsilon_{r} } \right)d\varepsilon = N_{t} \left\{ {1 - \exp \left[ { - \left( {\frac{{\varepsilon_{r} }}{{F_{0} }}} \right)^{m} } \right]} \right\} $$

With the increase of load, the internal damage of rock block continues to accumulate, micro-cracks appear and expand, and finally form macro cracks, resulting in the failure of a rock mass. The damage variable $D_{m}$ is the macro expression of rock micro-defects, with a value between 0 and 1, corresponding to the state that the rock loses its bearing capacity from initial loading to complete failure, substituting Eq. (21) into Eq. (19), the damage variable $D_{m}$ as Eq. (22):

22 $$ D_{m} = 1 - \exp \left[ { - \left( {\frac{{\varepsilon_{r} }}{{F_{0} }}} \right)^{m} } \right] $$

Under the action of triaxial compression load^34^, Hooke’s law is followed when the micro-element of the rock block is loaded, and the transverse isotropic damage constitutive model of the rock block (Fig. 1a) without considering the bedding deformation can be obtained, as Eq. (23):

23 $$ \varepsilon_{r} = \frac{{S\sigma_{r} }}{{\left( {1 - D_{m} } \right)}} = \frac{{(S_{13} \sigma_{x} + S_{23} \sigma_{y} + S_{33} \sigma_{r} )}}{{\exp \left[ { - \left( {\frac{{\varepsilon_{r} }}{{F_{0} }}} \right)^{m} } \right]}} $$

After the transformation of the damage statistical equation, it is not necessary to determine the number of micro-elements in Eq. (19). Expression of microscopic defects of rock block by macroscopic damage variable D This paper mainly discusses the macroscopic mechanical properties and damage evolution law of slate. For this purpose, the determination of damage parameters *F*_*0*_ and m in Eq. (23) should also be given.

#### Determination of damage parameters

In Eq. (23), *F*_*0*_ and m in the damage constitutive model are the statistic parameters that characterize the concentration degree and macroscopic mechanical strength of slate micro-elements, which is an inherent property. The common methods to determine the statistical parameters based on the stress–strain curve obtained by the triaxial compression test are a linear fitting method, peak point method, and inversion analysis method^34,36,38^. The three methods have their advantages and disadvantages. In this paper, the peak point method is selected. The solution process includes complex derivation, but the peak point of the test stress–strain curve is well simulated. The implicit constitutive Eq. (24) is derived to obtain Eq. (25)

24 $$ S_{13} \sigma_{x} + S_{23} \sigma_{y} + S_{33} \sigma_{r} = \varepsilon_{r} \exp \left[ { - \left( {\frac{{\varepsilon_{r} }}{{F_{0} }}} \right)^{m} } \right] $$

25 $$ \frac{{d\sigma_{r} }}{{d\varepsilon_{r} }} = S^{ - 1} {\text{exp}}\left[ {\left( {\frac{{\varepsilon_{r} }}{{F_{0} }}} \right)^{m} } \right]\left\{ {1 - m\left( {\frac{{\varepsilon_{r} }}{{F_{0} }}} \right)^{m} } \right\} $$

According to the geometrical characteristics of the stress–strain curve of slate specimen under triaxial compression test, ^43^ it can be known that the peak point $\frac{{d\sigma_{r} }}{{d\varepsilon_{r} }} = {0}$ and the vertical Eqs. (24) and (25) can be used as parameters in the damage constitutive equation of rock block under triaxial compression load, as Eq. (26):

26 $$ \left\{ \begin{gathered} m = {1/}\ln \left[ {\frac{{\varepsilon_{r} }}{{S{}_{33}\sigma_{r} }}} \right] \hfill \\ F_{0} = \varepsilon_{r} m^{ - 1/m} \hfill \\ \end{gathered} \right. $$

### Deformation constitutive equation of bedding structural plane

With the change in ion concentration, pH value, and temperature in groundwater, the physical and chemical reactions of micro-elements in slate are complex and changeable^44,45^. It is difficult to quantify the influence of water erosion on the strength and deformation ability of the bedding structural plane from the microscopic point of view. Phenomenological theory shows that the change of macroscopic physical properties of the layered structural plane can characterize its deterioration degree^46^. It is generally considered that the deterioration degree of closed elastic modulus and shear modulus is the same when axial deformation and shear deformation occur in the layered structural plane under compressive load.

The damage of the bedding structure plane of slate under the coupling action of water erosion and triaxial compression load mainly includes two parts: (1) Bedding damage caused by water erosion before loading; (2) damage caused by bedding closure deformation and shear deformation instability during loading. According to the stress–strain curve obtained from the test, the change in the elastic modulus of the bedding structural plane is easy to analyze and measure. Under the action of water environment erosion, the damage variables of the bedding structural plane are defined as Eq. (27):

27 $$ D_{c} = 1 - \frac{{E_{jc} }}{{E_{j0} }} $$

In Eq. (27), $D_{c}$ is the damage variable of water environment erosion; $E_{j0}$ represents the closed elastic modulus of rock joint structural plane in its natural state, and $E_{jc}$ represents the closed elastic modulus of the bedding structural plane of rocks eroded by the water environment.

When carrying out triaxial compression tests on slate specimens with a joint dip inclination of 0°, The shear slip deformation value of the bedding structural plane is 0. The small stress increment at the initial stage of loading corresponds to the large strain increment, that is, the initial stage of the stress–strain curve is relatively flat. Here, the measurement method of the closed elastic modulus $E_{j0}$ and $E_{jc}$ of the bedding structural plane is described. It is approximately considered that the axial deformation of the specimen at the initial stage of the stress–strain curve is mainly the closed deformation of the bedding structure. Therefore, the slope of the initial gentle stage of the stress–strain curve is approximately regarded as the closed elastic modulus of the bedding structural plane.

**Figure 3:**
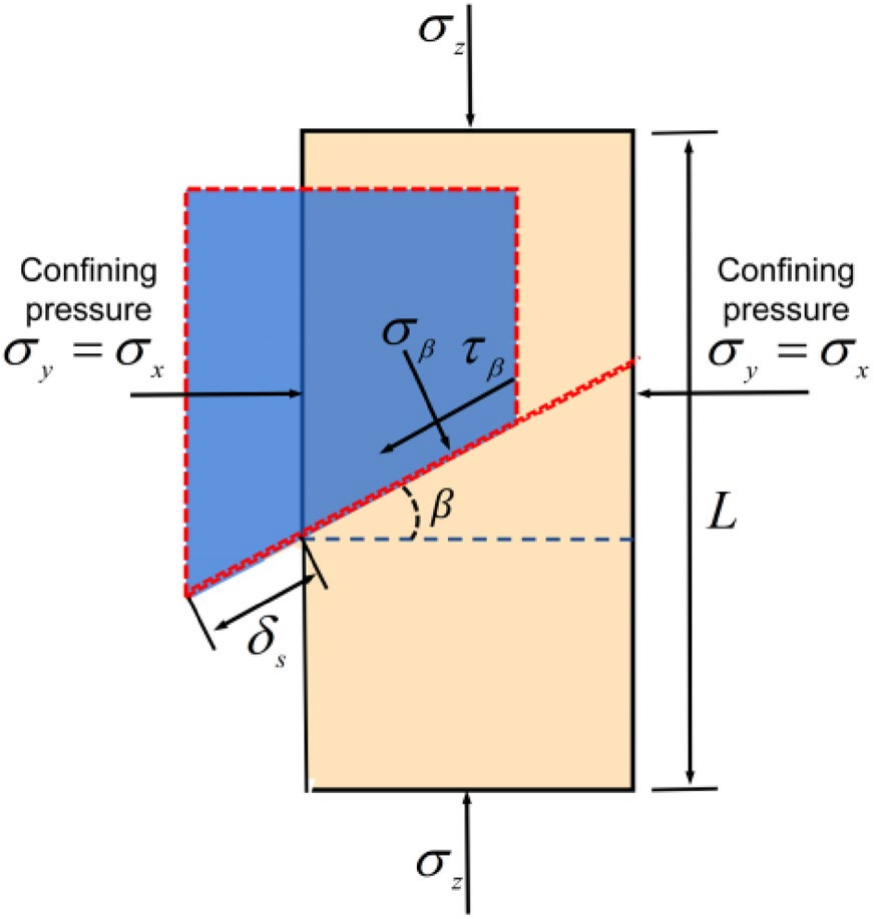
Diagram of triaxial compression stress of slate.

The deformation of the stratified slate under triaxial compressive loading includes the deformation of the rock block, the closing deformation of the bedding structural plane, and the shear slip deformation of the upper rock block along the joint plane. The mechanical model of laminated slate under triaxial compression load is shown in Fig. 3^40^. Under the action of three-dimensional stress^47^, the compressive stress and shear stress on the bedding structural plane of slate can be expressed by the Eq. (28):

28 $$ \left\{ \begin{gathered} \sigma_{\beta } = \sigma_{jc} \cos^{2} \beta + \sigma_{{\text{y}}} \cos \alpha \sin \beta \hfill \\ \tau_{\beta } = \sigma_{js} \cos \beta \sin \beta - \sigma_{{\text{y}}} \cos^{2} \beta \hfill \\ \end{gathered} \right. $$

The contribution of the closure deformation and shear deformation of the bedding plane to the axial deformation of the specimen is discussed, respectively.

First, consider the axial closure strain $\varepsilon_{jc}$ generated by the bedding structural plane, as shown in Eq. (29)^40,47^, and $\varepsilon_{j0}$ is the maximum closure strain of the structural plane. Under the triaxial compression load, the elastic modulus of the rock blocks containing micro defects is much larger than the closed elastic modulus of the bedding structural plane for the slate specimen with a joint dip angle of *β* = 0°. It is approximately considered that the strain value at the initial stage of the stress–strain curve is the maximum closed elastic modulus of the bedding structural plane.

29 $$ \left\{ \begin{gathered} E_{jc} = E_{j0} (1 - D_{c} ) \hfill \\ \varepsilon_{jc} = \varepsilon_{j0} \left[ {1 - \exp \left( { - \frac{{\sigma_{jc} \cos^{2} \beta + \sigma_{y} \cos \beta \sin \beta }}{{E_{jc} \varepsilon_{j0} }}} \right)} \right] \cdot \cos \beta \hfill \\ \end{gathered} \right. $$

Secondly, the shear slip of the bedding plane is considered to affect the total axial strain of the specimen. According to the constitutive relationship of bedding structural plane under shear load^39,46^, the deformation calculation expression is derived:

30 $$ \tau_{\beta } = K_{sc} \delta_{s} $$

In Eq. (30), $\tau_{\beta }$ is the shear stress on the joint surface; $K_{sc}$ represents the tangential stiffness of the joint surface of rock eroded by the water environment; $\delta_{s}$ is the shear displacement along the joint surface. The axial strain $\varepsilon_{js}$ of the specimen due to shear slip along the joint surface is as follows:

31 $$ \left\{ \begin{gathered} K_{sc} = K_{{s{0}}} (1 - D_{c} ) \hfill \\ \varepsilon_{js} = \frac{{\left( {\sigma_{js} \cos \beta \sin \beta { - }\sigma_{{\text{y}}} \cos^{2} \beta } \right)\sin \beta }}{{K_{sc} L}} \hfill \\ \end{gathered} \right. $$

In Eq. (31), $K_{s0}$ represents the tangential stiffness of the rock bedding structural plane in the natural state, and $K_{sc}$ represents the tangential stiffness of the rock bedding structural plane eroded by the water environment.

When the dip angle of bedding is 45°, the deformation of the layered structural plane under triaxial compression load includes closed deformation and shear slip deformation Similarly, in the initial stage of loading, the axial deformation of the specimen is approximately considered to be composed of the closure deformation and shear slip deformation of the bedding structural plane The shear modulus $K_{{s{0}}}$ and $K_{sc}$ can be calculated according to the slope of the initial stage of stress–strain curves of slate specimens with bedding dip angles of 0° and 45°.

In Eqs. (29) and (31), the damage variable of water erosion is given. The next step is to consider the damage evolution law of macroscopic mechanical properties of the bedding structural plane under triaxial compression load and give the calculation expression of damage coupling deformation of the bedding structural plane under compression load after water environment erosion. In this paper, the state of the bedding structural plane after water environment erosion is regarded as the initial state, and the damage state caused by compression load after water environment erosion is the second state. Under the action of triaxial compression load, it is considered that the damage deterioration law of the bedding structural plane is the same as that of rock block mechanical properties, and the coupling damage variable D of the bedding structural plane is expressed by the Eq. (32). Bring Eq. (32) into Eqs. (29) and (31) to obtain an Eq. (33) that can accurately describe the deformation of the bedding structural plane.

32 $$ D = ({1} - D_{m} )({1} - D_{c} ) = ({1} - D_{c} )\exp \left[ { - \left( {\frac{\varepsilon }{{F_{0} }}} \right)^{m} } \right] $$

33 $$ \left\{ \begin{gathered} \varepsilon_{jc} = \varepsilon_{j0} \left[ {1 - \exp \left( { - \frac{{\sigma_{jc} \cos^{2} \beta + \sigma_{y} \cos \beta \sin \beta }}{{{(1} - D_{c} )\exp \left[ { - \left( {\frac{\varepsilon }{{F_{0} }}} \right)^{m} } \right]E_{{j{0}}} \varepsilon_{j0} }}} \right)} \right]\cos \beta \hfill \\ \varepsilon_{js} = \frac{{\left( {\sigma_{js} \cos \beta \sin \beta { - }\sigma_{{\text{y}}} \cos^{2} \beta } \right)\sin \beta }}{{{(1} - D_{c} )\exp \left[ { - \left( {\frac{\varepsilon }{{F_{0} }}} \right)^{m} } \right]K_{{s{0}}} L}} \hfill \\ \end{gathered} \right. $$

### Establish triaxial compression damage constitutive model

Under the triaxial compression load, the axial deformation of laminated slate consists of three parts: the deformation of damaged rock mass containing micro-cracks, the closure deformation of the bedding structural plane, and shear slip deformation. In Sections “Damage variable of rock blocks containing micro defects” and “Deformation constitutive equation of bedding structural plane”, the detailed derivation process of the deformation calculation expression of the three components is given, respectively. Bring Eqs. (23) and (33) into Eq. (1) to obtain the triaxial damage constitutive model of laminated slate after water environment erosion under a triaxial compression load, as shown in Eq. (34).

34 $$ \left\{ \begin{gathered} \varepsilon_{r} = \frac{{(S_{13} \sigma_{x} + S_{23} \sigma_{y} + S_{33} \sigma_{z} )}}{{\exp \left[ { - \left( {\frac{{\varepsilon_{r} }}{{F_{0} }}} \right)^{m} } \right]}} \hfill \\ \varepsilon_{z} = \varepsilon_{r} + \varepsilon_{j0} \left[ {1 - \exp \left( { - \frac{{\sigma_{z} \cos^{2} \beta + \sigma_{y} \cos \beta \sin \beta }}{{(1 - D_{c} )\exp \left[ { - \left( {\frac{{\varepsilon_{{\text{r}}} }}{{F_{0} }}} \right)^{m} } \right]E_{{j{0}}} \varepsilon_{j0} }}} \right)} \right]\cos \beta {\kern 1pt} + \frac{{\left( {\sigma_{z} \cos \beta \sin \beta { - }\sigma_{{\text{y}}} \cos^{2} \beta } \right)\sin \beta }}{{(1 - D_{c} )\exp \left[ { - \left( {\frac{{\varepsilon_{r} }}{{F_{0} }}} \right)^{m} } \right]K_{{s{0}}} \cdot L}} \hfill \\ \end{gathered} \right. $$

The Eq. (34) contains three parts corresponding to the axial deformation of rock block under the coupling action of water environment erosion and triaxial compression load, the axial deformation caused by the closure deformation of the bedding structure plane, and the shear slip, respectively.

## Experimental work and results

To verify the accuracy and applicability of the damage constitutive model established in this paper, and explore the damage evolution law, failure characteristics, compressive strength, and deformation capacity of laminated slate after water environment erosion under triaxial compression load. The indoor triaxial compression experiments of layered slate samples with different confining pressures and bedding angles were carried out to analyze the effects of confining pressures and inclination changes on the damage evolution law, failure characteristics, compressive strength, and deformation capacity of the samples.

### Experiment preparation

**Figure 4:**
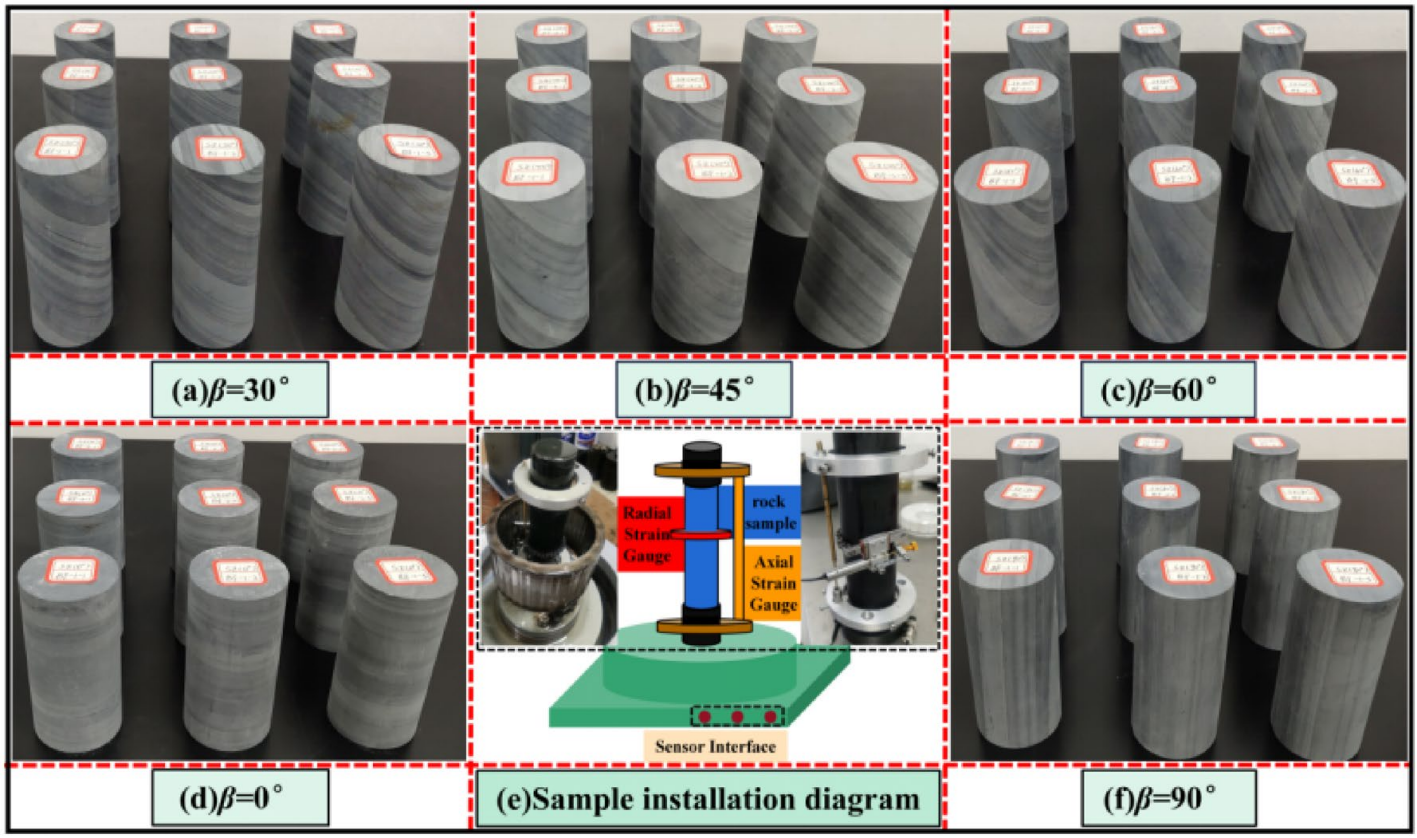
Layered slate samples with different dip angles.

To reduce the discrete type of rock samples and avoid the differences in composition and structure of rock samples, all slate samples are from the Bayue mountain tunnel of the Tongan expressway. According to the standards of the international society for rock mechanics (ISRM)^48^, the slate samples are processed into qualified samples. The flatness error of both ends of the specimen shall not exceed 0.02mm, and the side shall be smooth and straight, meeting the requirements of verticality, some rock samples are shown in Fig. 4.

To reveal the damage evolution mechanism of water-saturated laminated slate under triaxial compression load. Carry out triaxial compression tests of slate specimens after water environment erosion at confining pressures of 0 MPa, 5 MPa, 10 MPa, and 15 MPa. The statistics of the number of samples are shown in Table 2.

**Table 2:** Statistics of rock samples.

Confining pressure (MPa)	0°	30°	45°	60°	90°	Total
0	3	3	3	3	3	15
5	3	3	3	3	3	15
10	3	3	3	3	3	15
15	3	3	3	3	3	15
Total	12	12	12	12	12	60

**Figure 5:**
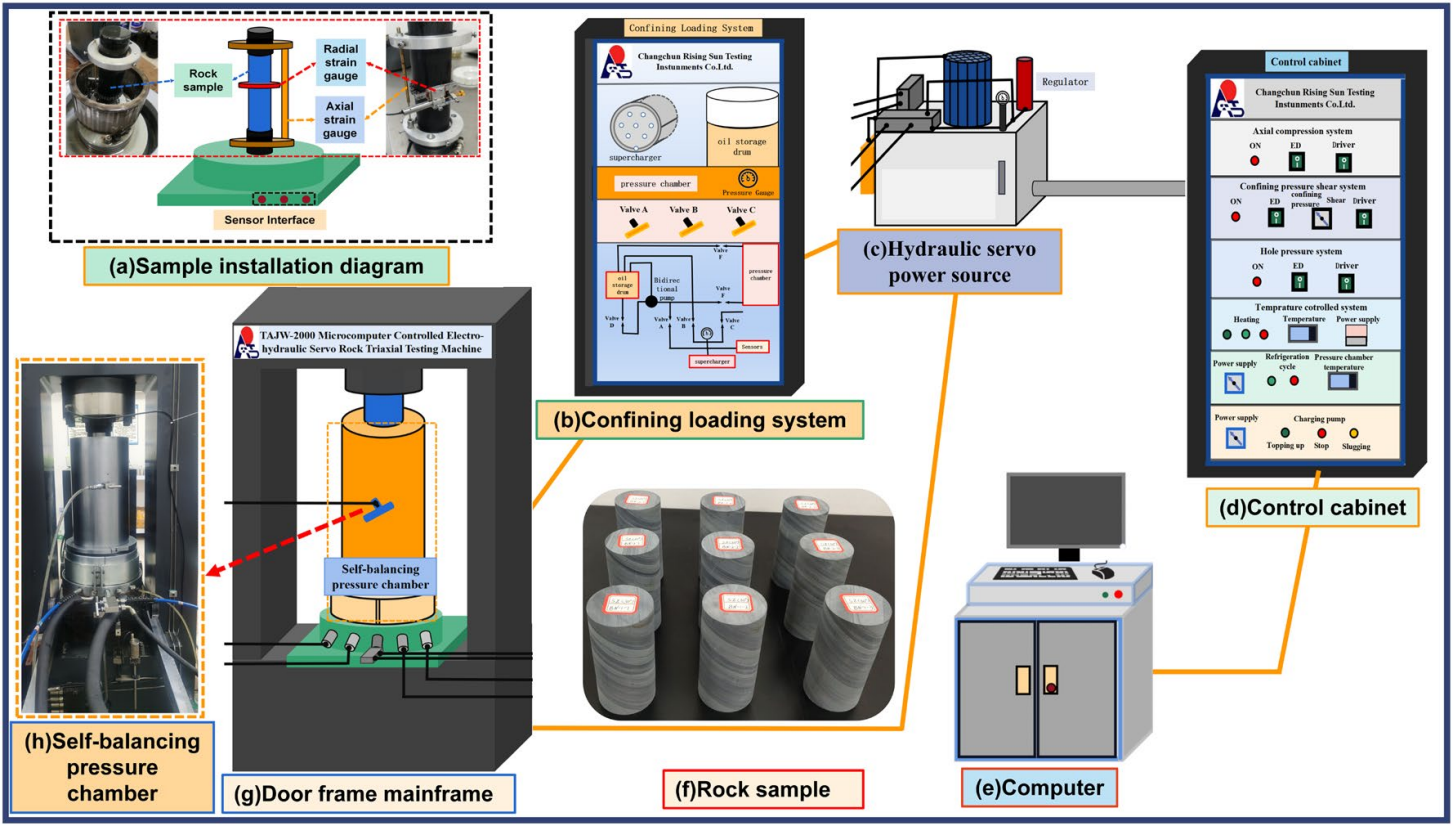
Microcomputer controlled electro-hydraulic servo rock triaxial test system.

The equipment used is a TAJW-2000 microcomputer controlled electro-hydraulic servo rock triaxial test system. Its loading control mode is equal displacement rate, and the loading rate is 0.005 mm/s, as shown in Fig. 5.The system consists of the main frame, hydraulic pump unit, controller, strain gauge, data acquisition module, and computer.

### Experimental results

#### Determine elastic parameters

Using 9 rock samples to carry out compression experiments with confining pressure of 0 MPa, five elastic parameters of transverse isotropy of rock blocks are determined, and the damage variables of closure modulus and shear modulus of bedding structural plane are obtained.

**Figure 6:**
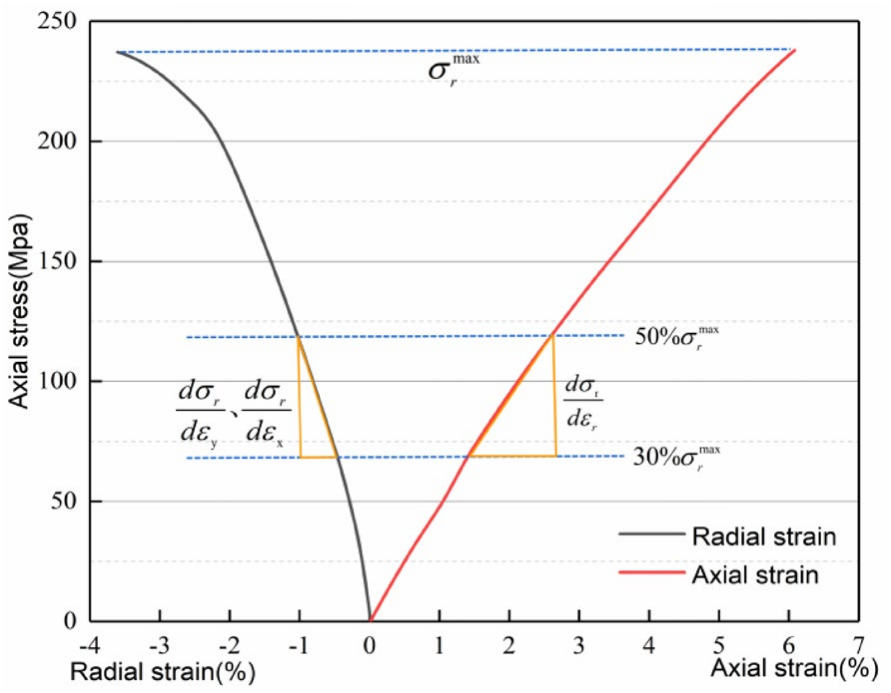
Stress strain curve of test piece.

The method to determine the five independent elastic parameters of slate is as follows. As shown in Fig. 6, take the reciprocal of the slope that is the stress–strain curve at 30–50% of the peak stress, and determine five independent elastic constants in combination with the equation given in Table 1.

In Eq. (33), the closing deformation and shear slip deformation of the bedding plane of the slate specimen change with the change of the angle when the bedding dip angle is different. Current the anisotropy angle* β* = 0°, cos*β* = 1, sin*β* = 0, the axial component value of shear slip deformation of bedding structural plane is $\varepsilon_{js}$ = 0, *E*_*2*_ and *v*_*2*_ can be solved by Eqs. (11) and (12); Current the anisotropy angle* β* = 90°, cos*β* = 0, sin*β* = 1, The axial component value of shear slip deformation of the bedding structural plane is $\varepsilon_{js}$ = 0, and the axial component value of closed deformation is $\varepsilon_{jc}$ = 0. The axial deformation of slate specimen is the axial deformation of Rock blocks containing micro defects, *E*_*1*_ and *v*_*1*_ can be solved by Eqs. (14) and (15); When the anisotropy angle 0° < *β* < 90°, the deformation of the specimen consists of three parts: the axial deformation of the rock block, the axial closure deformation, and shear slip deformation of the bedding structural plane, *G*_*2*_ can be solved by Eq. (18).The calculation results of the five independent elastic parameters are shown in Table 3:

**Table 3:** Five elastic parameters of the wet slate obtained from uniaxial tests.

Statistics	*E*_*1*_ (GPa)	*E*_*2*_ (GPa)	v_1_	v_2_	*G*_*2*_ (GPa)
Min	30.19	26.12	0.21	0.23	7
Max	46.94	34.74	0.23	0.28	12
Mean	37.24	29.33	0.22	0.25	9

According to the experimental test method given in Section “Deformation constitutive equation of bedding structural plane”, three slate specimens with a bedding dip angle of 0°and 45° after natural state and water environment erosion are selected to carry out the uniaxial compression test, respectively. The maximum closure strain of the structural plane $\varepsilon_{j0}$ = 0.00098, the closed elastic modulusof the joint surface $E_{j0}$ = 1973 MPa, $E_{jc}$ = 1712 MPa, the tangential stiffness of the joint surface $K_{sc}$ = 1727 MPa/m, the tangential stiffness of the joint surface $K_{s0}$ = 1989 MPa/m, the value of damage variable $D_{c}$ is 0.132.

After solving the five independent elastic parameters, bring the average value into Eqs. (7)–(9), and calculate the s component of the elastic flexibility matrix of slate with different bedding dip angles, the results are listed in Table 4.

**Table 4:** Elastic flexibility matrix components.

Anisotropy angle *β*	S_13_	S_23_	S_33_
0°	− 8.5237e−06	− 8.5237e−06	3.40948e−05
30°	− 7.86968e−06	− 1.4733e−05	3.84949e−05
45°	− 7.21566e−06	− 1.68027e−05	3.87582e−05
60°	− 6.56164e−06	− 1.4733e−05	3.48848e−05
90°	− 5.90763e−06	− 8.5237e−06	2.68745e−05

#### Stress–strain curve

**Figure 7:**
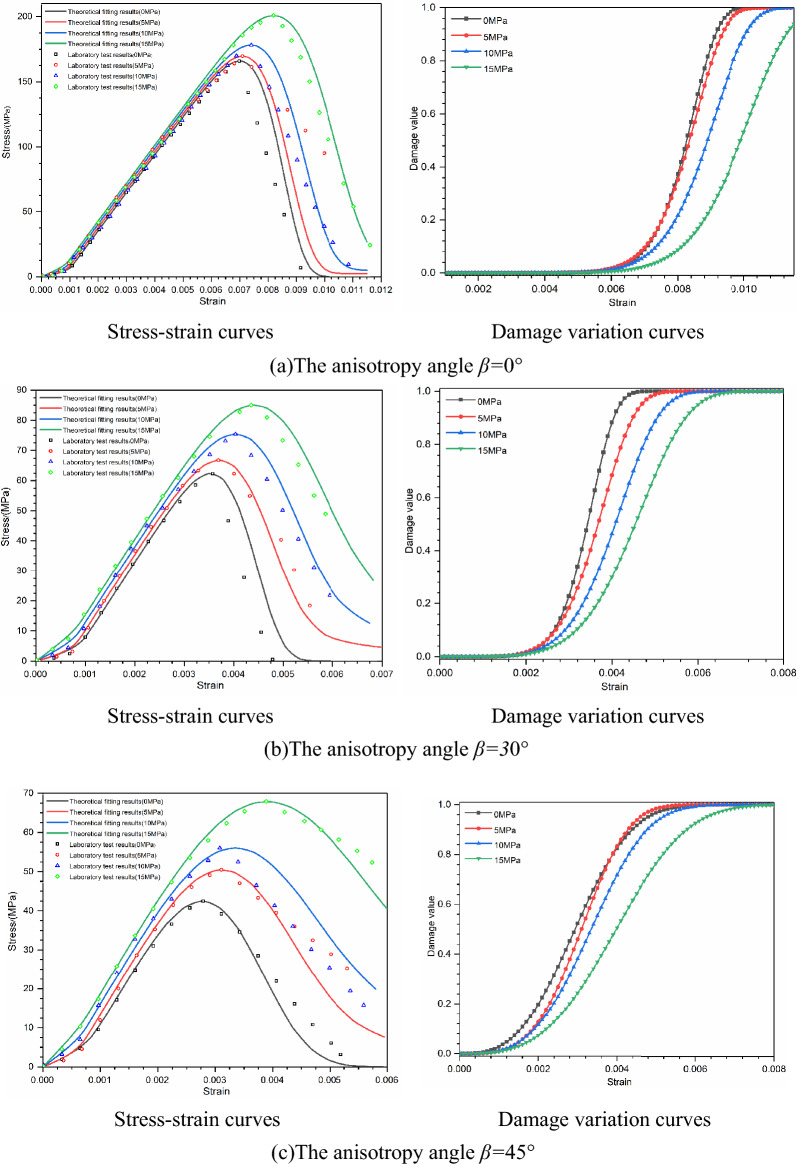

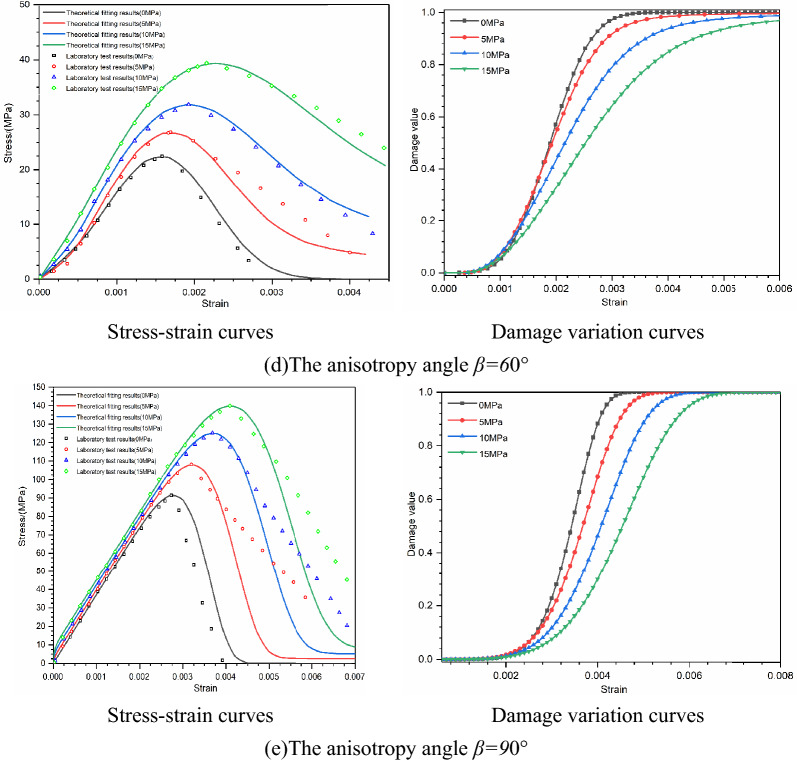
The stress–strain curves and damage variation curves of specimens under different bedding dip angles and confining pressures.

According to the above experimental scheme, the triaxial compression test of laminated slate is carried out, and the stress–strain relationship curve is obtained and compared with the constitutive model established in this paper. The Stress–strain curve and damage evolution curve can completely reflect the mechanical behavior of laminated slate under a triaxial compression load, as shown in Fig. 7. The rising section of the theoretical calculation curve of layered slate with different bedding dip angles is consistent with the indoor triaxial compression test curve, which can better fit the deformation process of rock before failure, it shows that it is more reasonable to consider the deformation of slab strata. There are still some deviations in the post-peak softening stage due to the influence of rock-hard brittleness. After the specimen reaches the peak stress, the instability failure suddenly occurs. The experimental test equipment has no rigid auxiliary device, and the falling section of the stress–strain curve decreases linearly and rapidly.

In Table 4, *S*_11_ and *S*_23_ are the confining pressure coefficients in the x and y directions, respectively. With the increase of bedding dip angle (0° ≤ *β* ≤ 90°), the values increase first and then decrease, and reach the maximum when *β* = 60°. *S*_33_ represents the coefficient of axial load. With the increase of bedding angle, the value decreases first and then increases, and it is the smallest when *β* = 60°. The change trend of its value is consistent with the change trend of compressive strength of the sample, as shown in Fig. 7.

The parameter *m* describes the strength distribution concentration of trace elements in the rock mass and reflects the plasticity and brittleness characteristics of the jointed rock mass. The larger the value is, the better the plasticity is, and 60° is the minimum value. The parameter *F*_0_ is the average value of the strength of trace elements in macro statistics, which reflects the strength of jointed rock mass, and the change rule of its value is consistent with the change rule of the compressive strength of the sample, and 60° is the minimum value.

In Fig. 7, the damage evolution curve of bedding slate shows an “S” type distribution law with the whole, which is composed of three segments, the first is a gentle rising segment, the second is a fast rising segment, and the last is a slow rising segment, which tends to 1. When the bedding dip angle is 0°–60°, the initial gentle rising section of slate with the same bedding dip angle becomes longer with the increase of confining pressure; With the increase of bedding dip angle, the initial gentle ascending section becomes shorter; With the increase in bedding dip angle, the steepness of the rising section decreases. When the bedding dip angle is 90°, the rule of damage evolution curve is the same as that of 0°, and the length of the gentle rising section and the steepness of the rapid rising section are between 0° and 30°. The changing trend of the damage curve of layered rock samples is related to many factors, such as bedding dip angle, stress state, failure characteristics, compressive strength, and deformation capacity.

#### Analysis of failure characteristics of specimens

**Figure 8:**
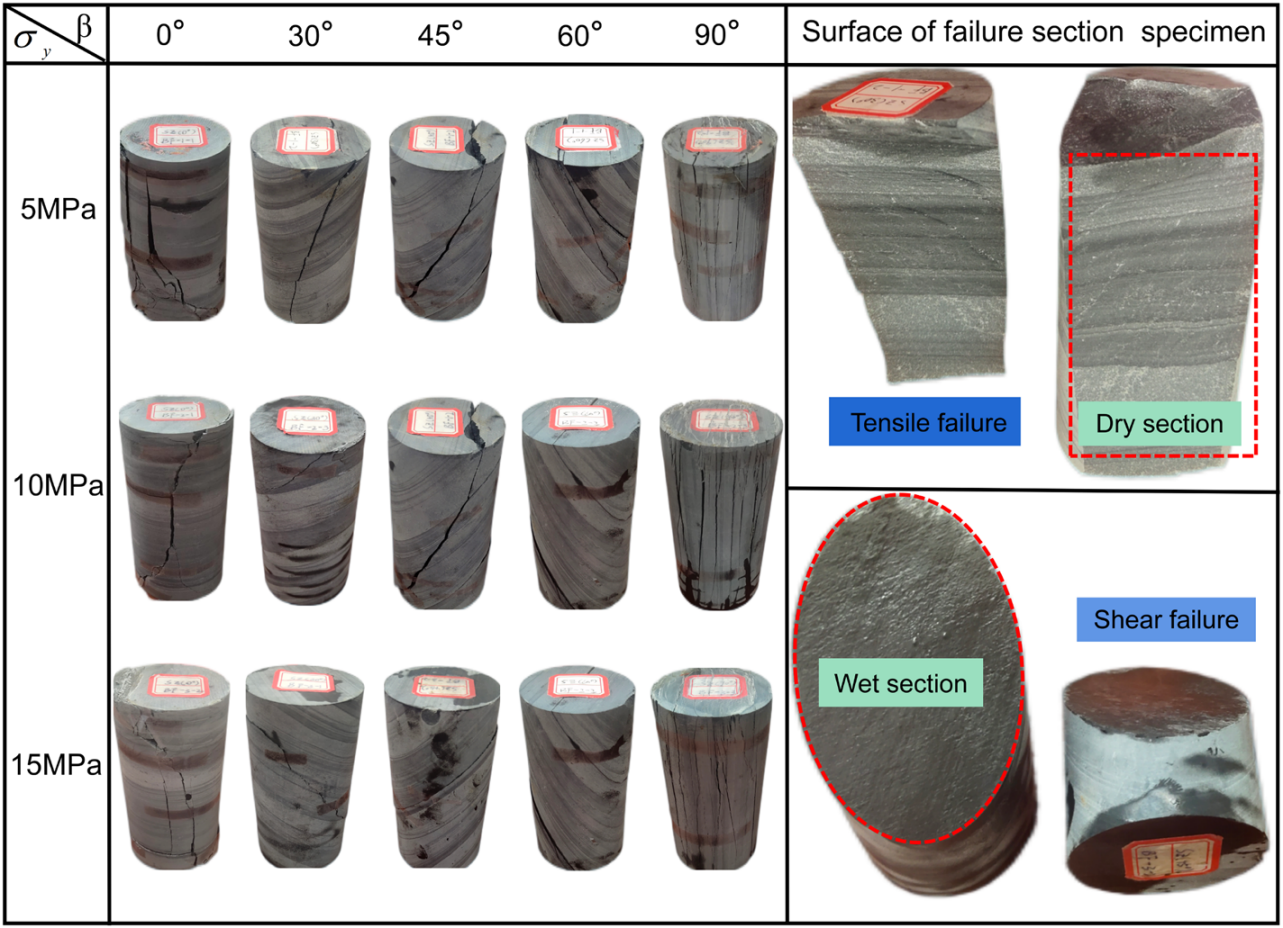
Failure diagram of specimen under different experimental conditions.

The layered slate shows obvious brittleness and anisotropy when it fails. Figure 8 shows the failure type and failure section morphology of slate specimen under compression load. The results show that confining pressure and bedding dip angle play a decisive role in the failure type of slate.

Current bedding dip *β* = 0°, the specimen has a tensile shear composite failure through the bedding plane, and the failure crack is wide. With the increase of confining pressure, the failure crack becomes narrow, and the failure type is mainly tensile failure. Current bedding dip *β* = 30°, the fracture surface is a composite failure of tension and shear along the bedding plane and through the bedding plane. With the increase of confining pressure, the crack develops in a stepped manner and expands fully. Current bedding dip *β* = 45°, the shear failure mainly occurs along the bedding plane, and the shear failure through the bedding occurs with the increase of confining pressure. Current bedding dip *β* = 60°, the shear slip failure occurred along the bedding plane, and the number of failure planes increased with the increase of confining pressure. Current bedding dip *β* = 90°, the specimen is fractured and tensioned along the bedding plane and parallel bedding plane. With the increase of confining pressure, the number of wide cracks leading to specimen failure increases, accompanied by more micro cracks.

In addition, it can be observed in Fig. 8 that when the failure type of the sample is mainly tensile, the failure section is dry, and the stratification of the failure section is significant; When the failure type of the specimen is shear slip failure along the bedding plane, the failure section is wet. It shows that when water environment erodes layered slate, the mechanical properties of its bedding structural plane will be greatly affected. Therefore, it is reasonable to consider the coupling effect of water environment erosion and compression load when establishing the damage constitutive model in this paper.

#### Analysis of compressive strength and deformation capacity

**Figure 9:**
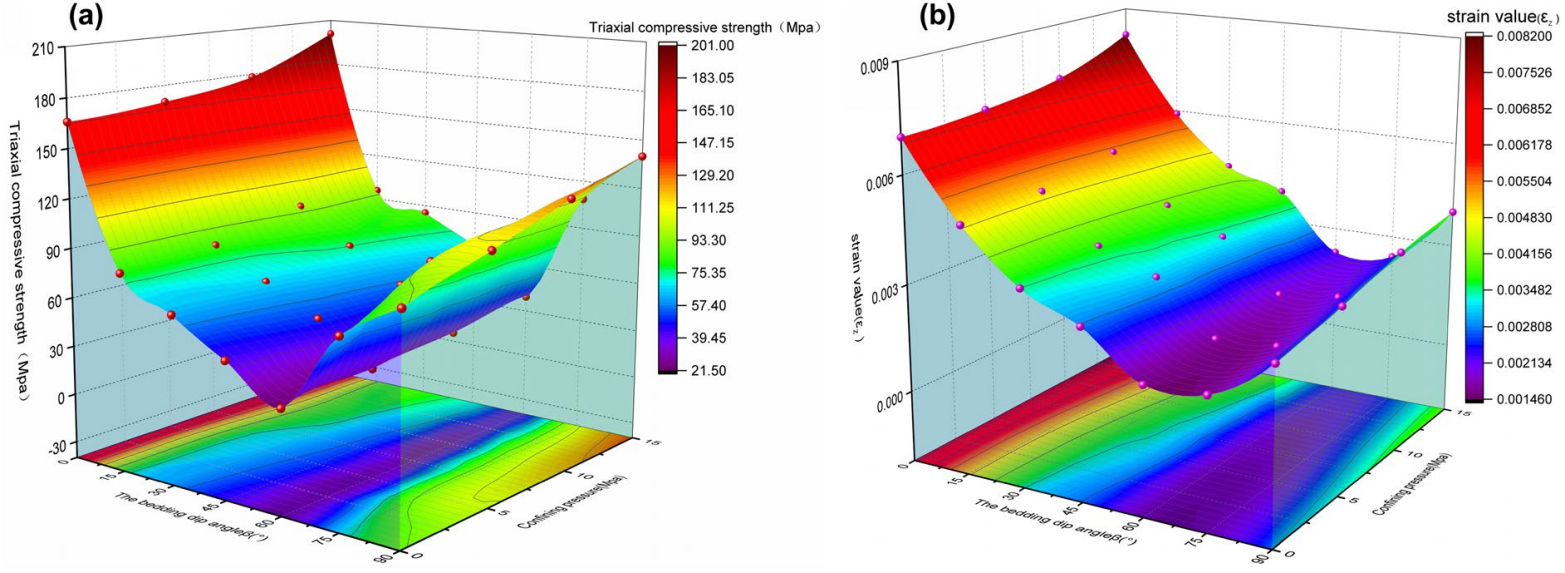
Two-factor change diagram of mechanical properties; (**a**) The variation of triaxial compressive strength of slate specimens with different bedding angles and confining pressures; (**b**) The variation of deformation capacity (strain value corresponding to peak stress) of slate specimens with different bedding angles and confining pressures.

The anisotropy of parameters characterizing mechanical properties is significant with the change of bedding dip angle. In Fig. 9a, under the same confining pressure, with the increase of bedding dip angle, the triaxial compressive strength of laminated slate first decreases and then increases, and the peak strength of the specimen presents a U-shaped parabola with the opening upward, which is consistent with much research results. When the bedding dip angle is 0°, the compressive strength of the slate is the largest, and the compressive strength of the slate with a bedding dip angle of 60° is the smallest. With the increase of confining pressure, the compressive strength of slate with the same bedding angle also increases, and the post-peak residual strength of the specimen is almost zero, with significant brittle failure characteristics.

In Fig. 9b, shows that the deformation capacity of the specimen is closely related to the compressive strength. It can be seen from Fig. 7 that at the beginning of loading, the stress–strain curves of slate samples with different bedding angles have a nonlinear compaction stage, and then the elastic deformation stage. When the bedding dip angle is 0°–45°, the compaction stage of the stress–strain curve of the rock sample is obvious, and the compaction stage becomes smaller with the increase of the dip angle; When the bedding dip angle is 60°, the compaction stage of the rock sample does not exist, mainly because the slate sample is mainly sheared along the bedding plane under a compression load, and the rock mass is relatively complete at this time; The compaction stage of the 90° rock sample is not obvious, mainly because the influence of the bedding structure of the sample on the compressive strength and deformation capacity is not considered. With the increase of confining pressure, the deformation capacity of slate with the same bedding angle increases, and the brittle failure characteristics are obvious.

## Conclusion

The damage constitutive model of slate under triaxial compression load is proposed, which includes the coupling damage variables of water environment erosion and compression load. The triaxial compression experiments of slate with different confining pressures and bedding dip angles were carried out to verify the accuracy and applicability. The influence of confining pressure and bedding dip angle on the damage evolution law, compressive strength, and deformation capacity of laminated slate after water environment erosion is given. The main conclusions are as follows:

1. It is assumed that the statistical law conforms to the Weibull distribution, and considering the influence of the erosion of the water environment, the change of confining pressure, and the bedding dip angle on the stress–strain relationship. The damage constitutive model of transversely isotropic slate under triaxial compression load is given, which is a concise expression that can accurately fit the compaction section and elastic section of the stress–strain curve.
2. The damage evolution curve of laminated slate is composed of three sections: gentle rising section, fast-rising section, and slow rising section. The overall distribution law is S-shaped. Confining pressure and bedding dip angle are important factors affecting the damage evolution law.
3. The triaxial compression test results show that when the bedding dip angle changes from 0° to 60°, the failure type of slate specimens develops from tension shear composite failure to shear slip failure; The specimens with a bedding dip angle of 90° undergo splitting tensile failure along the bedding plane and parallel bedding plane. In addition, with the increase of confining pressure, the number of main cracks increases when the slate specimen is damaged, and there are many micro-cracks.
4. With the change of dip angle, the compressive strength and deformation capacity of laminated slate change in a U-shape. When the bedding dip angle is 0°, the compressive strength and deformation capacity are the largest, and when the bedding dip angle is 60°, the compressive strength and deformation capacity are the smallest. With the increase of confining pressure, the mechanical properties of laminated slate are significantly improved, and the compressive strength and deformation capacity of specimens are improved.

## Data Availability

All data, models, or codes that support the fifindings of this study are available from the corresponding author upon reasonable request.
